# Evaluation of the Effect of the Fibroblast Growth Factor Type 2 (FGF-2) Administration on Placental Gene Expression in a Murine Model of Preeclampsia Induced by L-NAME

**DOI:** 10.3390/ijms231710129

**Published:** 2022-09-04

**Authors:** Margarita L Martinez-Fierro, Idalia Garza-Veloz, Maria Eugenia Castañeda-Lopez, Dorothy Wasike, Claudia Castruita-De la Rosa, Iram Pablo Rodriguez-Sanchez, Ivan Delgado-Enciso, Jose Flores-Mendoza

**Affiliations:** 1 Molecular Medicine Laboratory, Unidad Academica de Medicina Humana y Ciencias de la Salud, Universidad Autonoma de Zacatecas, Carretera Zacatecas-Guadalajara Km. 6, Ejido la Escondida, Zacatecas 98160, Mexico; 2 Faculty of Medicine, Comenius University Bratislava, Špitálska 24, 81372 Bratislava, Slovakia; 3 Laboratorio de Fisiologia Molecular y Estructural, Facultad de Ciencias Biologicas, Universidad Autonoma de Nuevo Leon, Monterrey 66450, Mexico; 4 School of Medicine, University of Colima, Colima 28040, Mexico; 5 Colima State Health Services, Cancerology State Institute, Colima 28040, Mexico

**Keywords:** preeclampsia, FGF2, angiogenesis, oxidative stress, L-NAME, gene expression, placenta

## Abstract

The abnormal implantation of the trophoblast during the first trimester of pregnancy precedes the appearance of the clinical manifestations of preeclampsia (PE), which is a hypertensive disorder of pregnancy. In a previous study, which was carried out in a murine model of PE that was induced by NG-nitro-L-arginine methyl ester (L-NAME), we observed that the intravenous administration of fibroblast growth factor 2 (FGF2) had a hypotensive effect, improved the placental weight gain and attenuated the fetal growth restriction, and the morphological findings that were induced by L-NAME in the evaluated tissues were less severe. In this study, we aimed to determine the effect of FGF2 administration on the placental gene expression of the vascular endothelial growth factor (VEGFA), VEGF receptor 2 (VEGFR2), placental growth factor, endoglin (ENG), superoxide dismutase 1 (SOD1), catalase (CAT), thioredoxin (TXN), tumor protein P53 (P53), BCL2 apoptosis regulator, Fas cell surface death receptor (FAS), and caspase 3, in a Sprague Dawley rat PE model, which was induced by L-NAME. The gene expression was determined by a real-time polymerase chain reaction using SYBR green. Taking the vehicle or the L-NAME group as a reference, there was an under expression of placental VEGFA, VEGFR2, ENG, P53, FAS, SOD1, CAT, and TXN genes in the group of L-NAME + FGF2 (*p* < 0.05). The administration of FGF2 in the murine PE-like model that was induced by L-NAME reduced the effects that were generated by proteinuria and the increased BP, as well as the response of the expression of genes that participate in angiogenesis, apoptosis, and OS. These results have generated valuable information regarding the identification of molecular targets for PE and provide new insights for understanding PE pathogenesis.

## 1. Introduction

Preeclampsia (PE) is a hypertensive syndrome of pregnancy (≥140 mmHg systolic or ≥90 mmHg diastolic) that occurs prior to the 20th week of gestation, which affects approximately 2–8% of women worldwide, contributing to both maternal and perinatal mortality and morbidity [1]. While the origin of PE has not yet been fully comprehended, it has been associated with the poor remodeling of the spiral arteries, whereby there is a decrease in the mean diameter of the external myometrial spiral artery [2]. Poor spiral remodeling by extravillous trophoblasts contributes to an alteration in the blood supply to the placenta, with subsequent ischemic episodes and changes in the oxygen supply in the maternal–fetal unit, triggering the wide variety of pathophysiological mechanisms that are associated with the clinical manifestations of PE [3].

In pregnancy, one of the vital processes during normal placental development is the formation of new blood vessel networks that enable the correct delivery of nutrients and oxygen to tissues from the mother to the fetus [4]. This process, which is called angiogenesis, is a complicated and well-ordered process that involves extensive signaling networks both between and inside the endothelial cells (ECs), the mural cells (the vascular smooth muscle cells and the pericytes), and other cell types (e.g., immune cells) [5,6,7]. Angiogenesis is regulated by a wide range of different angiogenic stimulators and inhibitors, and the normal endothelial cell turnover is the product of the correct balance between them [7]. During pregnancy, angiogenesis is promoted by vascular endothelial growth factor (VEGF) and placental growth factor (PLGF) [4], and it is modulated by antiangiogenic growth factors, such as soluble fms-like tyrosine kinase-1 (sFlt-1), which is a splice variant of Flt-1, and soluble endoglin (sENG), which is a truncated form of endoglin, both of which act as the antagonist of VEGF and PLGF and of transforming growth factor-β (TGFβ), respectively [8]. The appearance of the clinical manifestations of PE is related to the abnormal induction of the increased synthesis and release of sFlt-1 and sENG impairing their cell signaling pathways through distinct but additive mechanisms [8,9] and triggering placental oxidative stress (OS) [3]. Despite the efforts of the antioxidants, such as superoxide dismutase (SOD) and catalase (CAT), to ensure proper vascular functioning, the presence of placental ischemia reduces the anti-oxidative ability and enhances the OS [10]. Besides ischemia, hypoxia is one of the most powerful triggers for the increased production of VEGF and nitric oxide (NO), which induces both vasodilatation and angiogenesis [11]. NO is a known paracrine mediator that acts as a placental vasodilator and functions as a modulator during the ovulation, the implantation, the maintenance of pregnancy, the placental perfusion, the labor, and the delivery [3,12]. During the course of pregnancy, both placental angiogenic and oxidative imbalances, and/or an alteration in the bioavailability of NO, hinders normal pregnancy progression by disrupting the functioning of the placenta and contributing to the pathogenesis of PE [3,8].

Previous studies have stated the importance of fibroblast growth factor type 2 (FGF2) in the enhanced expression of VEGF [13]; furthermore, a relationship between a decreased circulating FGF2 concentration and PE development has also been reported [14]. In a previous study that was carried out in a Sprague Dawley rat model of PE that was induced by NG-nitro-L-arginine methyl ester (L-NAME), which is an inhibitor of nitric oxide synthase (NOS), we observed that the intravenous administration of recombinant FGF2 had a hypotensive effect, did not increase the maternal urine protein concentrations that are typically induced by L-NAME, improved the placental weight gain and attenuated the fetal growth restriction, and, histologically, the morphological findings that were induced by L-NAME in the evaluated tissues were less severe [12]. FGF family members have previously been noted to regulate the functioning of other growth factors, such as PLGF, monocyte chemoattractant protein 1, hepatocyte growth factor, and angiopoietin-2 [15,16,17]. This is consistent with our previous findings, whereby FGF2 was associated with the formation of the blood vessels [12]. Therefore, we hypothesize that these effects are due to the regulatory role of this growth factor in the well-known pathways that are associated with the pathophysiology of PE. Based on this, in this study we aimed to determine the effect of FGF2 administration on the placental gene expression of the key genes that are involved in angiogenesis, OS, and apoptosis, in a murine model that was induced by L-NAME.

## 2. Results

### 2.1. Modulatory Effect of FGF2 on BP Values and Urine Protein Concentration in the Rat PE-like Model Induced by L-NAME

**Figure 1:**
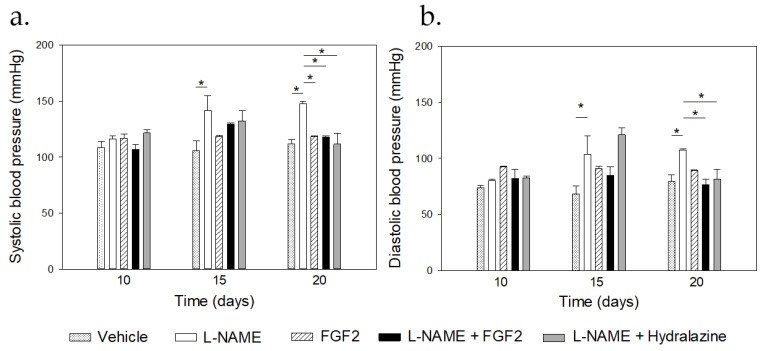
Comparisons of the blood pressure values between the treatment groups. Sprague Dawley rats (*n* = 3 for each group) were treated with vehicle, L-NAME (60 mg/kg/day), FGF-2 (666.6 ng/kg/day), L-NAME + FGF2, or L-NAME + hydralazine. Systolic (**a**) and diastolic (**b**) blood pressure values were recorded, and urine samples were collected from the 10th to the 20th day of pregnancy. * *p* < 0.05.

The PE-like model that has been used in this study was established in Sprague Dawley rats and it was induced using L-NAME at doses of 60 mg/kg/day, which were administered daily, starting on the 10th day of gestation until the 19th day of gestation, as previously reported [12]. Hypertension and proteinuria were the first two parameters that were considered as the reference for the establishment of the model [12]. In order to evaluate the modulatory effect of rhFGF2 on the BP values in the rat PE-like model, FGF2 was also administered daily (666.6 ng/kg/day), either alone or in combination with L-NAME. Figure 1 shows the BP measurements that were obtained for the groups of the vehicle, FGF2, L-NAME, L-NAME + FGF2, and L-NAME + hydralazine, on days 10, 15, and 20, respectively. Before the treatment, there were no significant differences in the BP values between the experimental groups (*p* > 0.05).

The mean of the SBP (Figure 1a) and the DBP (Figure 1b) before the treatment were 117.76 mmHg ± 1.9 and 74.7 mmHg ± 3.5 in the L-NAME group and were 116.5 mmHg ± 3.6 and 79.3 mmHg ± 6.7 in the vehicle group, respectively. There were differences in both the SBP and the DBP values between the L-NAME and the vehicle groups at day 15 (*p* = 0.001 for the SBP and *p* = 0.023 for the DBP) and at day 20 of pregnancy (*p* < 0.001). At days 15 and 20 of pregnancy, and compared with the vehicle group, there were no changes in the BP values in the groups that were treated with rhFGF2 alone or in the group that was treated with L-NAME + FGF2, relative to the vehicle group (*p* > 0.05). On day 20, there was a significant increase in the BP values in the L-NAME group when compared with the values that were observed in the vehicle, the FGF2, the L-NAME + hydralazine, and the L-NAME + FGF2 groups, respectively (*p* < 0.05). On day the 20th day of pregnancy, no differences in the BP values were observed between the vehicle and the L-NAME + FGF2 groups or between the vehicle and the FGF2 groups (*p* > 0.05).

**Figure 2:**
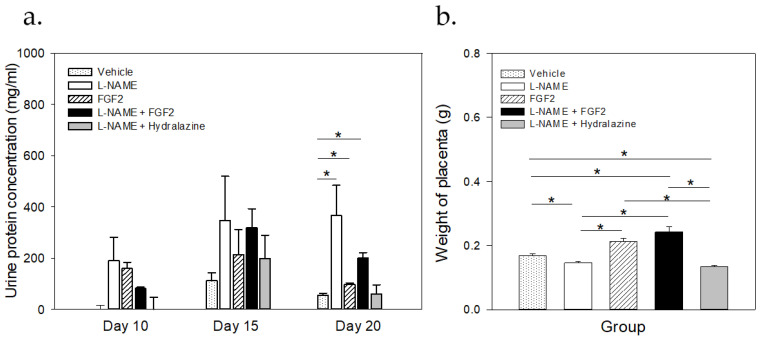
Comparisons of the urine protein concentrations and placental weight between the treatment groups. Sprague Dawley rats (*n* = 3 for each group) were treated with vehicle, L-NAME (60 mg/kg/day), FGF-2 (666.6 ng/kg/day), L-NAME + FGF2, or L-NAME + hydralazine (**a**). Urine proteins were quantified using the Bradford method. At the end of the protocol (20th day of pregnancy) placental tissue was collected (*n* = 6), weighed, and the mean of the weight was compared between groups (**b**). Data are represented as mean ± SE. * *p* < 0.05.

Figure 2a displays the results of the urine protein concentration for each experimental group on the 20th day of pregnancy. Before the treatment (from day 10 to day 15), there were no significant changes in the urine protein concentrations between the groups (*p* > 0.05). The normal urine protein concentration before the treatment ranged from 31.4 μg/mL to 154.8 μg/mL. On day 20 of pregnancy, there were differences in the urine protein concentrations between the L-NAME group and the vehicle, the FGF2, and the L-NAME + hydralazine groups (*p* < 0.05). At this time point, the urine protein concentrations of the L-NAME + hydralazine group decreased, reaching lower levels than those that were observed in the vehicle and the FGF2 groups. However, while there were differences in the urine protein concentrations between the vehicle and the L-NAME + FGF2 groups (*p* < 0.001), the urine protein concentrations in the FGF2 group did not differ from those that were observed in the vehicle group (*p* = 0.732).

### 2.2. FGF2 Administration Induced Changes in Placental Weight in the Rat PE-like Model Induced by L-NAME

After the experimental protocol was completed, on the 20th day of gestation, the placentas were collected and weighed. The results of these measurements are shown in Figure 2b. The mean weights of the placentas were 0.169 g ± 0.0299, 0.146 g ± 0.0231, 0.212 g ± 0.0495, 0.242 g ± 0.0748, and 0.134 g ± 0.0227, in the vehicle, the L-NAME, the FGF2, the L-NAME + FGF2, and the L-NAME + hydralazine groups, respectively (*p* < 0.05). When considering the L-NAME group as a reference, the weight of the placentas was significantly higher in the vehicle, the FGF2, and the L-NAME + FGF2 groups (*p* < 0.001). There were also differences in the placenta weight between the vehicle group and the L-NAME + FGF2 and L-NAME + hydralazine groups (*p* < 0.05), but not between the vehicle and the FGF2 groups alone (*p* > 0.05). There were no differences between the L-NAME and the L-NAME + hydralazine groups (*p* > 0.05).

### 2.3. FGF-2 Administration Modulated the Placental Gene Expression in the Murine PE-like Model Induced by L-NAME

**Figure 3:**
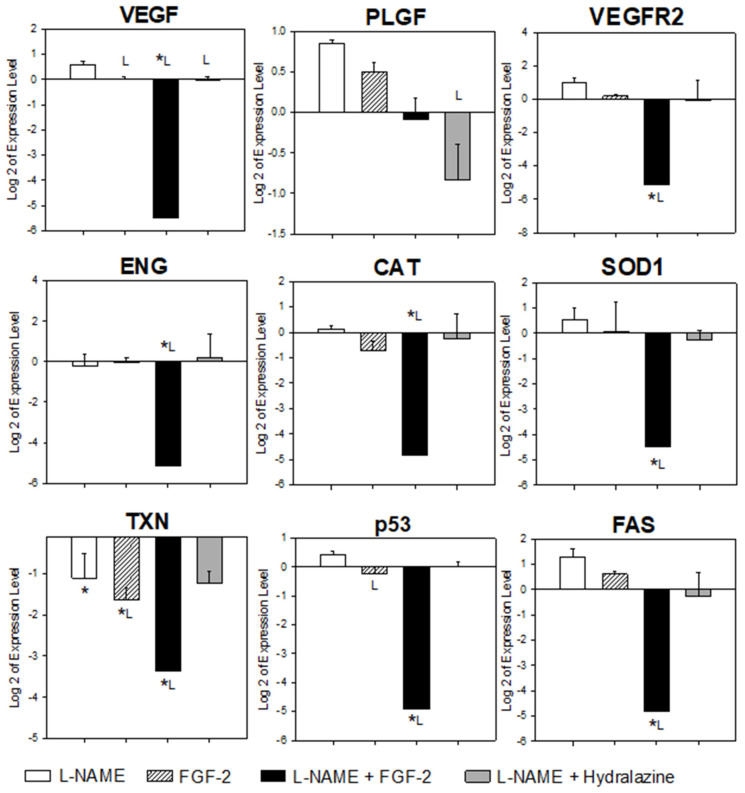
Comparison of placental gene expression between treatment groups. Sprague Dawley rats were treated with vehicle (NaCl 0.9%), L-NAME (60 mg/kg/day), FGF-2 (666.6 μg/kg/day), L-NAME + FGF2, or L-NAME + hydralazine. Placental samples (*n* = 6) were collected from three animals in each group on the 20th day of pregnancy. The expression levels of the genes involved in angiogenesis (VEGF, PLGF, VEGFR2, and ENG), oxidative stress (SOD1, CAT, and TXN) and apoptosis (p53 and FAS) were quantified by qRT-PCR, using SYBR Green, and HPRT1 as the endogenous gene. The data obtained from the vehicle group were considered as the calibrator during the gene expression calculations. One-way analysis of variance was carried out to compare each gene of interest between the treatment groups. Comparisons with *p*-values < 0.05 were subjected to multiple comparison analyses using the Holm–Sidak or Dunn’s method * *p* < 0.05 versus vehicle; L *p* < 0.05 versus L-NAME.

Figure 3 shows the results of the placental mRNA expression levels of the evaluated genes for each treatment group. Compared to the vehicle group, in the L-NAME group there was a significant under expression of TXN (*p* = 0.014). Although there was an apparent over expression of VEGF, PLGF, VEGFR-2, SOD1, p53, and FAS, and an under expression of ENG, these changes were not significant (*p* > 0.05). In the FGF2 group, only TXN showed a significant under expression (*p* = 0.01) when it was compared to the vehicle group. With the exception of PLGF, and taking the vehicle group as a reference, there was an under expression of all of the evaluated genes in the L-NAME + FGF2 group (*p* < 0.05). The L-NAME + hydralazine group showed an under expression of TXN when compared to the vehicle group (*p* = 0.017).

With the exception of PLGF, all of the evaluated genes were under expressed in the placentas from the rats in the L-NAME + FGF2 group when they were compared to the L-NAME group (*p* < 0.05). The VEGF was the only gene whose expression differed in the comparisons between the groups of the L-NAME and the FGF2, the L-NAME and the L-NAME + FGF2, and between the L-NAME and the hydralazine (*p* < 0.05). Compared with the L-NAME group, the p53 gene was also significantly under expressed in the FGF-2 group (*p* = 0.047).

Although the placental expressions of BCL2 and CASP3 were considered in this study, these genes were not included in the gene expression calculations or in the comparisons because the Cq values that were obtained for several placental tissues in the study groups were not observed in most of the animals and the amplification plots that were obtained had a Cq of > 38. Accordingly, and in order to avoid bias, these genes were excluded.

## 3. Discussion

Considering the previous evidence that the intravenous administration of FGF2 has beneficial and hypotensive effects, reducing the clinical manifestations of PE in a rat model [12], in this study we aimed to determine the effect of FGF2 administration on the placental gene expression of the key genes that are involved in angiogenesis, OS, and apoptosis, in a murine model that was induced by L-NAME.

Different working groups, including ours, have previously established the PE-like model that has been used in this study [12,18,19]. It is induced by the administration of L-NAME, which is an inhibitor of NO production, as it inhibits the NOS enzyme, prevents endothelium-dependent relaxation, and produces an increase in the BP [20]. As expected, during the establishment of the model, we observed a gradual increase in BP during the course of pregnancy and a hypotensive effect in the L-NAME group, which was derived from both the administration of the hydralazine, which is an agent that is known for its antihypertensive properties [21], and from the administration of FGF2, which may also regulate hypertension [12,22]. Similarly, besides hypertension, urinary protein excretion is a key finding during the pathogenesis of PE [23]. In this study, there was a decrease in the concentration of proteins in the urine from day 15 to day 20 of gestation in the L-NAME + FGF2 group; these results correlated with a decrease in the BP in the same group. It is well known that proteinuria causes hypoxia and the constriction of the uterine vessels, which may limit the supply of nutrients to the fetus, therefore, generating a low birth weight [24]. Similarly, previous studies have observed that the lack of endothelial synthesis of NO generates damage to the podocytes and L-NAME also causes global severe glomerular endotheliosis in rats, suggesting a beneficial decrement in the endotheliosis severity when FGF2 is administered [25]. These results suggests that FGF2 reduces the L-NAME-associated-injuries in the glomerulus, and it may also explain the decrement in the proteinuria that was observed in the L-NAME + FGF2 group in our study. Accordingly, the decrease in the BP and urine proteins after FGF2 administration represent a beneficial effect on both the mother’s health and on the growth of the offspring, as previously reported [12]. Additional studies are necessary in order to investigate the molecular mechanism by which FGF2 decreases renal damage, secondary to the L-NAME treatment.

Among the main molecular markers that are associated with the development of PE are those that are associated with angiogenesis, OS, and apoptosis, which are intimately involved in adequate cell proliferation, invasion and remodeling, and placental perfusion [26]. Similarly to the molecular mechanisms that are observed during PE pathogenesis, the administration of L-NAME in animal models, as seen with other NO inhibitors, also alters the different physiological mechanisms influencing endothelial regeneration, angiogenesis, apoptosis, and cellular OS [27,28]. In our study, compared to the vehicle group, there was no apparent impact of L-NAME administration on the placental mRNA quantity of the evaluated genes (with the exception of TXN). These results were unexpected; however, they seem to indicate that, in spite of the differences in both the placenta size and weight between the vehicle and the L-NAME groups, additional mechanisms exist that may compensate for the pathological process that is induced by L-NAME, which are reflected as subtle changes in the gene expression in the placenta at term. In humans, the placenta expression profiles of women with PE differed from those of women with normal pregnancies; however, the gene expression profiles have been widely variable between studies [29,30,31]. Although many factors could be responsible for these differences (e.g., the lack of consensus in the criteria to select the placental tissue for the experiments and the PE classification criteria) [29,30,31], it is probable that the placental pathological processes in both humans and mice, may have compensatory mechanisms that allow the pregnancy to end without any obvious differences in the expression profiles in the placenta at term.

The VEGF and its receptor, VEGFR2, are two important factors that collaborate in placental cell proliferation and angiogenesis during several stages of pregnancy [32]. Interestingly, in our study, when the L-NAME group was considered as the reference, there was a lower quantity of VEGF mRNA in the placentas from the FGF2, the L-NAME + FGF2, and the L-NAME+ hydralazine groups, with the difference being the largest in the L-NAME + FGF2 group (fold change = −9.5). Lower quantities of the mRNA of VEGFR2 were also found in the L-NAME + FGF2 group when they were compared to that observed in the placentas from the L-NAME group (fold change −8.8; *p* < 0.015). The placental under expression of VEGF that was observed in our study in the L-NAME group is consistent with that observed by Abe and collaborators, who reported that the VEGF mRNA expression in both rat placentas and placental explants was temporarily decreased by L-NAME treatment [33]. However, there are no reports on the effect of FGF2 administration on these genes in any animal model that were induced with L-NAME. In order to explain the modulation of VEGF/VEGFR2 expression in our study, we must consider the following additional previously reported findings: first, the inhibition of the NO generation by L-NAME results in a decreased VEGF synthesis [27]; second, the VEGF-dependent release of NO and the angiogenic activity of VEGFA is blocked by the action of L-NAME [27,33]; third, in tumors, if VEGF-dependent angiogenesis is blocked, FGF2-driven angiogenesis takes its place [34]. Accordingly, we propose that the under expression of VEGF in our study was a consequence of the treatment with L-NAME, but when FGF2 was continuously administered, the angiogenesis that was driven by FGF2 was triggered in order to compensate for the absence of the VEGF functions. Because the inhibition of tumor angiogenesis that was mediated by the VEGF signaling blockade with bevacizumab correlated with a reversal of VEGFR1 and VEGFR2 protein levels [35], it is highly probable that the under expression of VEGFR2 that was observed in our study may reflect a VEGF modulatory effect, since FGF2 is also capable of modulating the expression of VEGF and its receptors in both an autocrine and a paracrine way [36].

Antioxidant enzymes, such as CAT, SOD1, and TXN, intervene in the normal physiological processes that help the body to counteract the negative effects of OS. These molecules are involved in the development of the fetus, they play a role in the growth and development of the fetal–placental unit [37,38,39], and their expression is modulated depending on the month of gestation [40]. The abnormal regulation of the placental expression of the OS genes in women with PE is controversial because some authors have demonstrated their over expression [41,42]. Other reports have shown decreased levels of these enzymes in women with PE that are associated with IUGR and lipid peroxidation [43,44], while others have reported no difference in the placental levels of antioxidants in women with PE [42]. It has been postulated that the levels of OS-related genes may be influenced by the stage and the severity of the disease, as OS can initially upregulate the antioxidant enzymes, the level of which may decrease in the presence of more severe or prolonged stress [42]. Additionally, the down-regulation of the antioxidant enzymes, such as CAT, correlate with high levels of H_2_O_2_, which is involved in the activation of the signaling pathways that induce the proliferation, the migration, and the cell invasion of cancer cells [45]. In our study, the placental gene expression of the CAT and SOD1 genes was significantly down-regulated in the L-NAME + FGF2 group, but not in the group that was treated with L-NAME or with FGF2 alone. Our results are similar to those that were reported by Tang and colleagues in alveolar epithelial cells, who observed that FGF-2 attenuated inflammation and reduced OS and apoptosis by activating the PI3K/Akt signaling pathway [46]. Accordingly, we propose that the under expression of these genes requires both the inhibition of NO synthesis (NOS inhibition) and the activation of the FGF2 signaling pathways at the same time, which may reflect a compensatory mechanism because of its proliferative and angiogenic properties (which may still be active in the placentas from this group). TXN, in turn, plays an important role in the NO pathway by reducing its production and promoting its degradation [47]. In our study, the placental expression of TXN was found to be modulated by L-NAME and FGF2 alone, or in combination, and their effect on the TXN under expression was additive. When compared with the L-NAME group, the TXN mRNA showed a fold change of 1 × 10^−18^ in the vehicle group, −1.4 in the FGF2 group, and −3.0 in the L-NAME + FGF2 group. These results indicate that, in extreme conditions, such as the inhibition of the NO signaling pathway and its biological consequences, and also during the constant activation of the FGF2 route (which is associated with cell proliferation, cell migration, and angiogenesis, among others) the TXN system does not play a principal role in counteracting the OS and, therefore, is down-regulated. In agreement with this observation, in humans, the expression of the placental TXN was reduced in patients with PE [42,48].

Apoptosis is a key function in the cells that manage the induction of cell growth arrest. The FAS and p53 are involved in this pathway and are expressed in the decidua, the chorionic villi, the cytotrophoblast, and the syncytiotrophoblast, indirectly promoting the maintenance of pregnancy [49,50,51]. In our study, both of the genes were under expressed in the placentas from the rats that were treated with L-NAME + FGF2. The increase in the p53 expression corresponds to the hypoxia that is generated in the placenta by PE, at least in vitro [52], and has implications in the intrauterine growth restriction [53]. The FAS, on the other hand, is involved in mediating the maternal immune response, in cell remodeling, and in cell proliferation [51,54]; therefore, the increase in this protein is a direct indicator of placental apoptosis, which is a common finding in PE [55,56]. Although these findings are not completely comparable with ours, the under expression of the apoptosis-related genes may be due to the fact that the placental biopsies that were used in this study came from the rats that were at the end of their pregnancy. At this final pregnancy stage, the apoptosis is not expected to be an active process, and similarly to the OS genes, the p53 and the FAS under expression in the L-NAME +FGF2 group may be related to the chronic activation of the FGF2 signaling pathways, which is compatible with the presence of less apoptotic activity.

The study’s limitations are as follows: In this study, we determined the effect of FGF2 administration on the placental gene expression of the key genes in angiogenesis, OS, and apoptosis; although the existence of regulation at the transcription level was not necessarily reflected at the protein level, a study perspective will be to validate our results at the protein level. Similarly, the placental gene expression that has been evaluated in the study was only at the end of the rat gestation and, therefore, the evaluation of the FGF2 effect during the previous stages of pregnancy at the placental levels and on the other organs or tissues should be assessed in futures studies.

## 4. Materials and Methods

### 4.1. Ethical Approval

The trial was approved by the Ethics and Biosafety Committee of the Area of Health Sciences of the Universidad Autónoma de Zacatecas Francisco García Salinas in Zacatecas, Mexico, and it was registered with the following identification number: CEB-ACS/UAZ.Ofc.002/2015. All of the experimental procedures were carried out in accordance with the recommendations of the “Technical specifications for the production, care and use of laboratory animals”, Mexican guidelines (NOM-062-ZOO-1999).

### 4.2. Animal Treatment

We followed the experimental protocol that was described in detail in a previous study, carried out by Martinez-Fierro et al. 2021 [12]. Briefly, pregnant 10-week-old Sprague Dawley rats were separated into the following five treatment groups: Group 1. The vehicle group, which was administered with 0.9% of NaCl by the intragastric route, using a cannula and syringe of 4 mm diameter. Group 2. The FGF2 group, which consisted of pregnant rats treated with rhFGF2 (Sigma-Aldrich, St Louis, MO, USA), intravenously administered (666.6 ng/kg/day) using the tail vein (caudal). Group 3. The L-NAME group, which was administered with L-NAME (NG-nitro-L-arginine methyl ester; Sigma-Aldrich, St Louis, MO, United States of America) by the intragastric route at a concentration of 60 mg/kg/day. Treatments in the vehicle group, FGF2 group, and L-NAME group began on the 10th day of gestation and concluded on the 19th day of pregnancy. Group 4. The L-NAME + FGF2 group, which consisted of pregnant rats that were administered daily both L-NAME and rhFGF2 simultaneously, as described above, beginning on the day 10th up to the 19th day of pregnancy. Group 5. The L-NAME + hydralazine group, in which L-NAME was administered, as described above, along with oral hydralazine at 10 mg/mL/kg/day by the intragastric route from the 15th to 19th day of pregnancy.

### 4.3. Biological Samples and Data Collection

The blood pressure (systolic: SBP, and diastolic: DBP) and urine protein levels were quantified and recorded on the 10th, 15th, and 19th days of pregnancy. The placental tissues were collected on the 20th day of gestation from each animal in the experimental groups. Each tissue was weighed and then all of the tissues (approximately 0.5 cm^3^/each) were embedded in Tissue-Tek^®^ O.C.T™ Compound (Sakura Finetek, Torrance, CA, USA). The embedded tissues were preserved by freezing at −80 °C until use.

### 4.4. Placental RNA Isolation and cDNA Synthesis

The frozen placental tissues that were stored at −80 °C and embedded in Tissue-Tek^®^ O.C.T™ Compound were defrosted at room temperature and were separated from the preservative. Total RNA was isolated from homogenized placental tissue, according to the RNeasy Mini Kit protocol (QIAGEN, Hilden, Germany). The RNA concentration and its purity were measured via optical density at 260 nm and the relation 260/280, respectively, using a NanoDrop 2000 spectrophotometer (Thermo Fisher Scientific, Wilmington, DE). The cDNA was synthesized from 1 mg of the total RNA using a High-Capacity cDNA Reverse Transcription Kit and random hexamers (Thermo Fisher Scientific, Wilmington, DE, USA), according to the manufacturer’s instructions. The cDNA samples were stored at −20 °C until use.

### 4.5. Gene Selection and Primer Design

The study genes were selected according to their relationship with the angiogenesis, apoptosis, and OS process. The genes selected included the following: VEGFA, PLGF, vascular endothelial growth factor receptor 2 (VEGFR2), ENG, tumor protein P53 (P53), BCL2 apoptosis regulator (BCL2), Fas cell surface death receptor (FAS), caspase 3 (CASP3), SOD1, CAT, and thioredoxin (TXN). In addition, the hypoxanthine phosphoribosyltransferase 1 (HPRT1) gene was selected as a reference gene. All gene-specific primers for the real-time qPCR assay were independently designed (Table 1) and provided by T4OLIGO^®^ (T4OLIGO, Guanajuato, Mexico).

### 4.6. Quantitative Real-Time Polymerase Chain Reaction (qRT-PCR)

Quantitative real-time PCR (qRT-PCR) was carried out using a StepOne Plus Real-Time PCR System (Applied Biosystems, Foster City, CA, USA) in 96-well PCR plates. Fifty nanograms of synthesized cDNA were used as templates for qRT-PCR amplification in a 10 μL of final reaction volume, using SYBR™ Green PCR Master Mix (Thermo Fisher Scientific, Wilmington, DE, USA), and 300 nM gene specific primers. The amplifications were performed with the following thermal cycle program: pre-denaturation for 10 min at 95 °C, amplification of 40 cycles with denaturation for 15 s at 95 °C, and annealing for 1 min at 60 °C. The cycle series were followed by a melt-curve analysis to confirm the specificity of amplification and the lack of primer dimers. All samples were analyzed in duplicate, including two non-template controls, to detect any template contamination. The 2^−ΔΔCq^ equation was applied to calculate the relative expression of the placenta samples [57]. The mean of quantification cycle (Cq) of the vehicle group samples was used as a calibrator. The sequences and product sizes of the forward and reverse primers for all of the evaluated genes are listed in Table 1.

### 4.7. Statistical Analysis

All data were expressed as mean ± standard error (SE) for three animals per group. Comparisons between two groups of data were carried out by Student’s *t*-test. For multiple comparisons of data, one-way analysis of variance (ANOVA), coupled with the Holm–Sidak method, was used; for non-normally distributed variables, the Kruskal–Wallis ANOVA on ranks, and the Dunn’s method as a multiple comparison procedure was applied. One-way repeated measures ANOVA, coupled with the Holm–Sidak test as a post hoc test, was used to evaluate whether there were differences in the BP values and urine protein concentrations within the same experimental group during the evaluated times. All statistical analyses were carried out using Sigma Plot^®^ version 11 (Systat Software Inc., San Jose, CA, USA). A 95% confidence interval (CI) was used and *p* < 0.05 was considered statistically significant.

## 5. Conclusions

In conclusion, we have corroborated that the administration of FGF2 in a murine PE-like model that was induced by L-NAME reduces the effects that are generated by proteinuria and increased BP. In presence of NOS inhibition, the intravenous administration of FGF2 during pregnancy induced lower quantities of placental mRNA of the VEGFA, VEGFR2, ENG, P53, FAS, SOD1, CAT, and TXN genes, in the model that was evaluated here. These results demonstrate that the pathogenic consequences of NOS inhibition that are induced by L-NAME during pregnancy may be modulated by FGF2 and are reflected as placental under expression of genes that are related to angiogenesis, apoptosis, and OS, thus, generating valuable information for the identification of molecular targets for PE, and for understanding the complex pathogenesis of PE.

## Figures and Tables

**Table 1:** General characteristics of the primers designed for the genes included in the study.

Gene Symbol	Gene Bank ID	Description	* Primer Sequence (5′→3′)	Tm	Product Size (bp)
VEGFA	NM_031836.3	Vascular Endothelial Growth Factor A	Fw: GGAGCAGAAAGCCCATGAAGTGGT Rv: TCATTGCAGCAGCCCGCACA	65	168
VEGFR2	NM_013062.2	Vascular Endothelial Growth Factor Receptor 2	Fw: TTTGCACTGCAGGAGCGCGT Rv: GGAATCGCCAGGCAAACCCACA	65	171
ENG	NM_001010968.3	Endoglin	Fw: CAGGGCTTCGTACAGGTGAGCA Rv: TCACACAGCTGCCCTTGGCT	64	139
P53	NM_030989.3	Tumor Protein P53	Fw: GTTGCTCTGATGGTGACGGCCT Rv: ACCACCACGCTGTGCCGAAA	65	112
BCL2	NM_016993.2	BCL2 Apoptosis Regulator	Fw: TCCAGGATAACGGAGGCTGGGATGC Rv: AGGCTGAGCAGCGTCTTCAGAGACA	67	103
FAS	NM_139194.3	Fas Cell Surface Death Receptor	Fw: GTCAACCGTGTCAGCCTGGTGAA Rv: TGGGTCCGGGTGCAGTTCGTTT	65	190
CASP3	NM_012922.2	Caspase 3	Fw: GCGGAGCTTGGAACGCGAAGAAA Rv: TCCAGAGTCCATCGACTTGCTTCCA	65	120
SOD1	NM_017050.1	Superoxide Dismutase 1	Fw: TTCGTTTCCTGCGGCGGCTTCT Rv: GGTTCACCGCTTGCCTTCTGCT	66	169
CAT	NM_012520.2	Catalase	Fw: GGCACACTTTGACAGAGAGCGGA Rv: TGAGCCTGACTCTCCAGCGACT	65	184
TXN	NM_053800.3	Thioredoxin	Fw: TCTGCCACGTGGTGTGGACCTT Rv: ACAGTCTGCAGCAACATCCTGGC	66	126
PLGF	NM_053595.2	Placental Growth Factor	Fw: TGAGGAACCCCACCTGTGATGCT Rv: CATTCAGCAGGGACGAGTTGGCT	65	156
HPRT1	NM_012583.2	Hypoxanthine phosphoribosyltransferase 1	Fw: CAGTCCCAGCGTCGTGATTA Rv: TGGCCTCCCATCTCCTTCAT	60	168

* All of the primer sequences were designed based on the respective GenBank sequence for the examined gene and between exons to guarantee the specific detection of the interest gene.

## Data Availability

Data that support the findings of this study are available from the corresponding author, upon reasonable request.
